# Computational Prediction of Single-Domain Immunoglobulin Aggregation Propensities Facilitates Discovery and Humanization of Recombinant Nanobodies

**DOI:** 10.3390/antib14030073

**Published:** 2025-08-28

**Authors:** Felix Klaus Geyer, Julian Borbeck, Wiktoria Palka, Xueyuan Zhou, Jeffrey Takimoto, Brian Rabinovich, Bernd Reifenhäuser, Karlheinz Friedrich, Harald Kolmar

**Affiliations:** 1 Institute for Organic Chemistry and Biochemistry, Technical University of Darmstadt, Peter-Grünberg-Strasse 4, 64287 Darmstadt, Germany; 2 GIP AG, xyna.bio, 55131 Mainz, Germany; 3 Drug Discovery and Development, Fuse Biotherapeutics, Woburn, MA 01801, USA; 4 Centre for Synthetic Biology, Technical University of Darmstadt, 64283 Darmstadt, Germany

**Keywords:** nanobodies, immunoglobulin domains, protein engineering, protein aggregation, AI-based structure prediction

## Abstract

Background/Objectives: Single-domain immunoglobulins are small protein modules with specific affinities. Among them, the variable domains of heavy chains of heavy-chain-only antibodies (VHH) as the antigen-binding fragment of heavy-chain-only antibodies (also termed nanobodies) have been widely investigated for their applicability, e.g., therapeutics and immunodiagnostics. However, despite their advantageous biochemical and biophysical characteristics, protein aggregation throughout recombinant synthesis is a serious drawback in the development of nanobodies with application perspectives. Therefore, we aimed to develop a computational method to predict the aggregation propensity of VHH antibodies for the selection of promising candidates in early discovery. Methods: We employed a deep learning-based structure prediction for VHHs and derived from it likely biophysical and biochemical properties of the framework region 2 with relevance for aggregation. A total of 106 nanobody variants were produced by recombinant expression and characterized for their aggregation behavior using size exclusion chromatography (SEC). Results: Quantitative characteristics of framework region 2 patches were combined into a function that defines an aggregation score (AS) predicting the aggregation propensities of VHH variants. AS was evaluated for its capability to forecast recombinant VHH aggregation by experimentally studying VHH Fc-fusion proteins for their aggregation. We observed a clear correlation between the calculated aggregation score and the actual aggregation propensities of biochemically characterized VHHs Fc-fusion proteins. Moreover, we implemented an easily accessible pipeline of software modules to design nanobodies with desired solubility properties. Conclusions: AI-based prediction of VHH structures, followed by analysis of framework region 2 properties, can be used to predict the aggregation propensities of VHHs, providing a convenient and efficient tool for selecting stable recombinant nanobodies.

## 1. Introduction

Nanobodies are highly versatile and robust single-domain antibodies. They can be used in a wide variety of applications [1], ranging from diagnostic applications like non-invasive imaging in vivo [2] to therapeutic utilization like in the case of Caplacizumab for the treatment of acquired thrombotic thrombocytopenic purpura [3]. They are characterized by favorable biophysical properties, such as high solubility, thermostability, and low aggregation tendency [4]. Nevertheless, during processes such as humanization and affinity maturation, aggregation can be a problem that affects rapid and efficient development [5,6,7]. Even though the variable domain of the heavy chain of heavy-chain-only antibodies (VHHs) usually has a high sequence similarity to the human variable domain of heavy chains (VHs), there are some strongly conserved differences, such as the highly conserved exchange of hydrophobic to hydrophilic residues in framework region 2 (FR2) compared to human VHs. In human VHs, FR2 participates in the VH:VL interface by interacting with the variable domain of the light chain (VL). In contrast, the FR2 region of VHHs is exposed to the solvent, and hydrophobic residues are replaced with more hydrophilic residues to increase solubility. These exchanges concern V42, G49, L50, and W52, which are mostly exchanged for F, E, R, and G/F. A few VHHs, about 10%, have a VGLW motif [5,8,9,10]. Conversion of the hallmark residues FERF/FERG in FR2 often leads to impaired stability and expression [6,11]. Human VHs that lack a light chain are usually prone to aggregation [12,13]. It has been shown that engineering human VHs by changing the hallmark residues to camelid equivalents improves their solubility and stability without a light chain [12,14]. These findings support the hypothesis that FR2 plays a central role in VHH aggregation. Additionally, in about 10% of all VHHs, W118, which is also part of the FR2 interdomain contact area, is exchanged for an Arg as a consequence of an unusual D-J recombination [8,15]. This exchange was proposed to enhance solubility and to improve stability in some cases [11,16,17]. This residue is also located within the conserved immunoglobulin domain interface [11,18]. Another notable feature of most VHHs is an elongated Complementarity-Determining Region 3 (CDR3) loop that is proposed to shield the FR2 interface and, thereby, prevent aggregation [19]. Moreover, it is often stabilized by an additional non-canonical disulfide bond with either CDR1 or CDR2, depending on the respective camelid species. This additional disulfide bridge has been suggested to prevent aggregation [20].

To predict the aggregation tendencies of given proteins in general, AGGRESCAN3D 2.0 has been introduced to estimate protein aggregation propensities based on structural information [21]. While it provides useful solubility estimates, it does not explicitly consider the critical contribution of the FR2 region to nanobody aggregation. Predicting biophysical properties, such as aggregation, remains a challenging task, and general tools like AGGRESCAN3D 2.0 are designed for a broad range of proteins, which can limit their specificity for specialized proteins such as VHHs. Here, we focus on the solubility characteristics of immunoglobulin domains. As we hypothesize that the conserved FR2 region of VHH domains represents a primary determinant of aggregation potential in the context of nanobodies, the goal of this work is to develop a sequence-based method explicitly based on the FR2 region characteristics for the prediction of nanobody aggregation propensities. By focusing on the distinctive features of VHHs, this approach offers a strategy that complements existing general-purpose aggregation prediction tools.

We employed a deep learning-based structure prediction as the foundation for analyzing key aggregation determinants in the FR2 region, condensed into a scoring function to estimate aggregation based on the VHH sequence. We recently pursued the development of VHHs with various specificities and application potential [22,23]. Candidate nanobodies were humanized by grafting the CDRs onto human frameworks with simultaneous random variation of the Vernier residues that are known to direct the spatial orientation of CDRs, followed by library screening for functional variants. The experimental findings on aggregation behavior from these campaigns, extended by the results on nanobodies for which quantitative aggregation data exist, were correlated with the calculated aggregation scores. Although focusing on only a small structural segment of the VHH, the outcome of this process was superior in distinguishing aggregation-prone from soluble nanobodies to the AGGRESCAN3D process, which covers the structure of the entire protein.

Taken together, applying the presented aggregation score to just the FR2 regions of VHHs allows for the prediction of their aggregation propensity from their respective sequences in a rational and fast manner. This facilitates VHH development by excluding aggregation-prone candidates from further studies, optimizing nanobody discovery, e.g., during high-throughput screening of nanobody libraries obtained from animal immunization.

## 2. Materials and Methods

### 2.1. Generation of VHH Libraries, Enrichment, and Selection of Binders by Fluorescence-Activated Cell Sorting (FACS)

To obtain VHH-based binders from immune libraries, Bactrian camels were immunized with the corresponding antigen. Yeast surface display (YSD) collections were generated for the identification of candidate VHHs. The number of independent transformants for the immune libraries was in the range of 10^7^–10^9^. In addition, synthetic YSD libraries for VHH humanization and affinity maturation were generated by overlap extension PCR. The theoretical diversities were below 10^8^, and the number of transformants was also between 10^7^ and 10^9^. Yeast library generation was conducted according to Benatuil et al. [24]. In total, three Bactrian camels and one alpaca were immunized with five to six different antigens each. Bactrian camel 1 was immunized with ROR1, and, subsequently, ROR1 binding VHHs were selected by phage display, as described in Zhou et al. [22]. For Bactrian camels 2 and 3 and alpaca 1, cDNA was synthesized using the extracted RNA. DNA coding for VHHs was enriched, as described in [25], and used for the generation of yeast surface display libraries, which were sorted by fluorescence-activated cell sorting (FACS). The libraries were subsequently enriched for binding over several rounds using FACS. Single clones were tested individually for antigen binding by flow cytometry. DNA from yeast clones was isolated using Zymoprep Yeast Plasmid Miniprep I (Zymo Research, Irvine, CA, USA, Cat No: D2001) and retransformed into *E. coli*, followed by plasmid isolation using the Wizard^®^ Plus SV Miniprep DNA Purification System (Promega, Madison, WI, USA, CAT No: A1460) and Sanger sequencing (Microsynth, Balgach, Switzerland).

### 2.2. Protein Preparation, Size Exclusion Chromatography (SEC) Analysis, and Quantification of Aggregation

Codon-optimized DNA constructs were synthesized by BioIntron (Shanghai, China) and cloned as VHH-hinge-CH2-CH3 fragments into a pCDNA3.4 mammalian expression IgG vector downstream of a CMV promoter. The constructs were sequence-verified and transfected into ExpiCHO cells (Thermo Fisher Scientific, Waltham, MA, USA, Cat. No. A14527). The cells (6.0 × 10^6^) were mixed with 3.5 mL of homemade BioIntron electrolysis solution and plasmid, electroporated, and cultured in 100 mL of OPM medium (Cat No. P93059) at 37 °C, 120 rpm, 8% CO_2_. Sodium butyrate was added after 24 h, and the culture continued for 6 days. Recombinant proteins were purified via protein A chromatography (VDOBIOTCH, Suzhou, China, Cat No. HQ320827001L) and assessed for monomer purity via SEC. An SEC analysis was conducted using an AKTA Pure 25 M1 protein purification system (GE Healthcare, Chicago, IL, USA) with a Superdex 200 Increase 10/300 GL column (GE Healthcare, Chicago, IL, USA Cat No: 28-9909-44). The column was soaked with 50 mL of distilled water and equilibrated with 50 mL of mobile phase buffer (50 mM sodium phosphate, 150 mM sodium chloride, pH 7.0) at 0.5 mL/min. Testing proteins, diluted to 25 µL in mobile phase buffer, were loaded onto the column, and the elution was monitored spectrometrically at 280 nm. The area under the curve (AUC) was determined for the different peaks in the chromatogram, and the fraction of monomeric protein was quantified. For normalization, the behavior of one particular VHH showed complete precipitation, and no measurable remaining soluble protein was exploited. The monomeric species value for this VHH was set to 0%.

### 2.3. Humanization of VHHs

Complementarity-determining regions (CDRs) of VHH clones were delineated using a statistical approach for VHH-specific loop identification, as described by Melarkode Vattekatte et al. [26]. To support rational humanization while preserving antigen-binding function, Vernier residues—framework positions known to modulate the conformation of CDR loops [27]—were identified based on structural homology to characterized VHHs and informed by the established literature [11,28,29]. These residues were considered critical for maintaining the native paratope geometry upon framework substitution. The closest human germline variable heavy (VH) framework was determined via sequence alignment using IgBLAST [30]. Human germline sequences with the highest identity to the camelid VHHs were selected as templates for defining permissible amino acid substitutions at each Vernier site. Residues were chosen to balance sequence humanization with preservation of VHH-specific features, particularly in framework region 2, where hydrophilic camelid-specific residues contribute to solubility and folding [31,32]. Degenerate oligonucleotides encoding the designed amino acid diversity were used to generate humanized VHH libraries by PCR assembly. Yeast surface display (YSD) was employed to express the libraries in *Saccharomyces cerevisiae*, taking advantage of eukaryotic secretory pathway processes including disulfide bond formation and chaperone-assisted folding [33]. Functional selection of properly folded and antigen-binding variants was carried out through iterative rounds of fluorescence-activated cell sorting (FACS), allowing the enrichment of clones with favorable binding and expression profiles.

### 2.4. In Silico Data Processing and Model Generation

Sequence Numbering and Alignment:

The amino acid sequences of VHH domains were numbered and aligned using the IMGT scheme [34] via ANARCI (version 2024.05.21) [35]. Residues 39–55 were defined as framework region 2 (FR2), and residue 118 as the first position of framework region 4 (FR4). Collectively, these residues were designated the “former VH:VL interface”.

Structure Prediction: Structural modeling of the VHHs was performed using NanoBodyBuilder2 [36], accessed through an online interface (https://opig.stats.ox.ac.uk/webapps/sabdab-sabpred/sabpred/nanobodybuilder2/ (accessed on 11 July 2025)). The modeling used ImmuneBuilder version 1.1.1. The predicted structures were downloaded in a PDB format for downstream analyses.

Structural Visualization and Feature Extraction: The modeled structures were analyzed using Mol* Viewer (v3; https://molstar.org/viewer/ (accessed on 11 July 2025)) [37], focusing on residues constituting the former interface (FR2 and residue 118).

### 2.5. Accessible Surface Area (ASA)

ASA values for residues 39–55 and 118 (*Ai*) were obtained using Mol*’s Residue Properties feature, which computes ASA using the Shrake–Rupley algorithm [38] using the default parameter settings (radius: 1.4 Å; n-point: 100). The visualization tool allows for residue-level interrogation by color-coding surface exposure and showing the ASA value for selected residues. Each ASA value was multiplied by the corresponding hydropathy index based on the Wimley–White scale (*Hi*) [39]. The mean ASA-weighted hydropathy score was calculated.

Hydrophobic Side-Chain Interaction Analysis: Intramolecular hydrophobic side-chain interactions (*hi*) were analyzed using Mol* Viewer [34], with a cutoff distance of 4 Å for defining interactions. For selected residues, the number of hydrophobic interactions was determined as the number of unobstructed paths between hydrophobic atoms, as described by Sehnal et al. [37]. The mean number of hydrophobic interactions per residue at the former VH:VL interface was then calculated.

Instability Index Calculation: The instability index for FR2 (residues 39–55) was computed using Expasy ProtParam (https://web.expasy.org/protparam/ (accessed on 11 July 2025)). This index estimates protein stability by assessing the frequency of destabilizing dipeptides and was used here as a proxy for local destabilization.

Automation of Aggregation Score Calculation: The described manual analysis pipeline was later automated using the xyna.bio platform and is available for free academic use with full documentation under xyna.bio/nanobodyAS.

Sequence Cluster Analysis: FR2 cluster analysis was performed using MMseqs2 via the MPI Bioinformatics Toolkit (version: c552cce6c3194c06bc0bba84f04c4ef13d62f0a5). The analysis was conducted with a minimum sequence identity of 0.8, a minimum alignment coverage of 0.9, and the --slow-sensitive mode enabled [38,39,40]. Clusters containing fewer than four sequences were discarded. Sequence logos were generated using WebLogo, version 2.8.2 [41].

### 2.6. Statistics

Unless stated otherwise, all statistical analyses were performed using GraphPad Prism (version 10.1.0; GraphPad Software, San Diego, CA, USA). To assess the homogeneity of variances among groups with differing sample sizes, Levene’s test was employed. In cases where the assumption of equal variances was met (*p* > 0.05), the Kruskal–Wallis test (a non-parametric alternative to one-way ANOVA) was used to evaluate differences in group medians. This approach was chosen due to the presence of certain groups’ deviations from normal distribution characteristics. For post hoc pairwise comparisons, Dunn’s multiple comparison test was applied to control for family-wise error rates and provide adjusted *p*-values. This method allows for the robust evaluation of intergroup differences, accounting for both group size and number. The statistical significance was defined as *p* < 0.05 for all analyses.

Receiver operating characteristic (ROC) curves were generated using GraphPad Prism. Precision–recall curves and the Youden index J were calculated using MedCalc^®^ (version 23.3.5; MedCalc Software Ltd., Ostend, Belgium).

The *accuracy* was calculated as follows:

$$accuracy=\;\frac{true\;positive\;\left(TP\right)+true\;negative\;(TN)}{positive\;\left(P\right)+negative(N)}$$

*Balanced accuracy*, which provides a more reliable measure for imbalanced datasets, was calculated as follows:

$$balanced\;accuracy=\;\frac{sensitivity+specificity}{2}$$

where

$$sensitivity=\frac{true\;positive\;(TP)}{true\;positive\;\left(TP\right)+false\;negative}$$

$$specificity=\frac{true\;negative\;(TN)}{true\;negative\;\left(TN\right)+false\;positive\;(FP)}$$

*Fβ* values were calculated as follows:

$$F\beta =\;\frac{\left(1+\beta^{2}\right)*true\;positive\;(TP)}{\left(1+\beta^{2}\right)*true\;positive\;\left(TP\right)+false\;postive\;\left(FP\right)+\beta^{2}*false\;negative\;(FN)}$$

For the recall-focused analysis (*β* > 1), a value of *β* = 2 was used, while for the precision-focused analysis (*β* < 1), *β* = 0.5 was applied.

## 3. Results

### 3.1. Characterization of VHH-Targeting ROR1 and Determination of Their Aggregation Behavior

**Figure 1:**
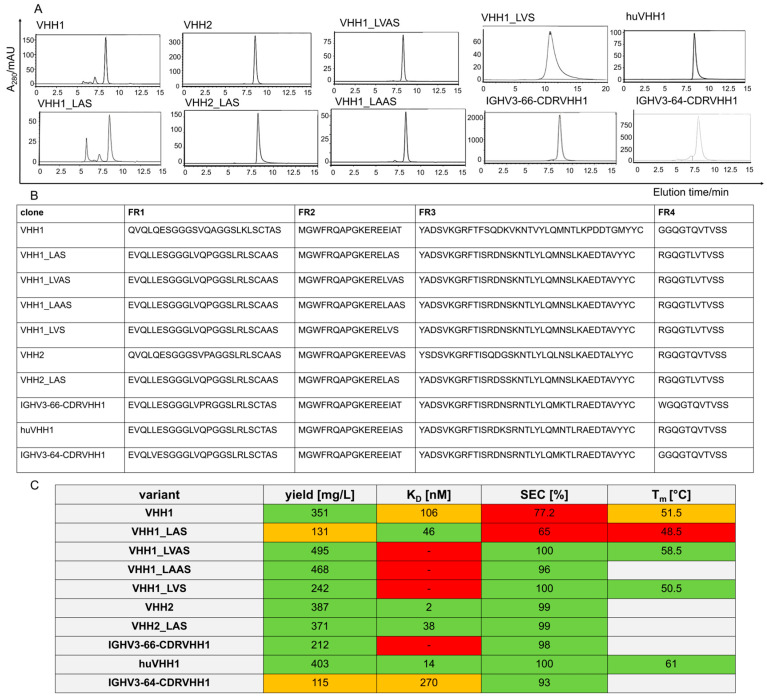
Size exclusion chromatography (SEC) analysis of VHHs expressed as Fc-fusions and purified by protein A chromatography. (**A**) SEC chromatograms of different VHHs. Analyzed were the camelid clones VHH1 and VHH2, along with their corresponding humanized versions using the IGHV3-23 framework with a deletion in FR2 (VHH1_LAS and VHH2_LAS). Additionally, variants with reinserted residues (VHH1_LVAS, VHH1_LAAS) and a point mutation (VHH1_LVS) were included. Humanized clones using the IGHV3-66 and IGHV3-64 frameworks (IGHV3-66-CDRVHH1, IGHV3-64-CDRVHH1, and huVHH1) were also analyzed. Absorbance was detected at 280 nm. (**B**) Sequences of the framework regions of the different VHHs are listed according to IMGT numbering. (**C**) Overview of the biophysical properties of the expressed VHH Fc-fusions, including yield (mg/L), dissociation constant (KD) for ROR1 binding measured by BLI, percentage of monomeric species in SEC, and melting temperature (Tm) determined by a protein thermal shift assay using SYPRO Orange. Suitable biophysical properties are highlighted in green, mediocre properties in orange, and unsuitable properties in red.

The starting point for this study was a project aimed at the discovery and humanization of nanobody-targeting ROR1 (Receptor Tyrosine Kinase-like Orphan Receptor 1). After immunization of a Bactrian camel with the IG-like domain of human ROR1, specific VHHs were isolated, as described in work by Zhou et al. [22]. The two parental clones, VHH1 and VHH2, which possessed an additional non-canonical disulfide bridge between CDR1 and CDR3, were chosen for humanization. They were reformatted for expression as Fc-fusions, which were subsequently purified by protein A chromatography. The parental clone VHH2, expressed as an Fc-fusion, did not significantly form aggregates. In contrast, VHH1 VHH showed about 23% aggregation in the size exclusion chromatography (SEC) analysis (Figure 1). For humanization, the human framework IGHV3-23 was used, and synthetic libraries were generated with allowed back mutations to the camelid residues at the Vernier positions [27]. Binding candidates were enriched using fluorescence-activated cell sorting (FACS).

After humanization of VHH1, only one functional clone was identified. This clone, VHH1_LAS, retained target binding but showed increased aggregation. Unexpectedly and not intentionally introduced during humanization, VHH1_LAS has a deletion in the framework region 2 (FR2) at position 53. For further specifying aggregation determinants, we reinserted a valine residue, which is found in human germline IGHV3-23, or introduced an alanine residue at the deleted position. The resulting VHHs, VHH1_LAAS and VHH1_LVAS, did not aggregate upon recombinant production. The same applies to a variant in which the original alanine 54 in FR2 has been replaced by a valine, VHH1_LVS. Interestingly, the deletion framework applied to the VHH2 (VHH2_LAS) clone showed no aggregation. Likewise, when using IGHV3-66 as a different human acceptor framework for humanization of VHH1, the resulting clone (IGHV3-66-CDRVHH1) with CDRs grafted showed no aggregation. A clone (huVHH1) using the same IGHV3-66 framework for Vernier residue optimization and affinity maturation by CDR randomization also showed no aggregation. In conclusion, this set of variants only differs from each other by one deletion and/or a few amino acid exchanges in framework residues while displaying a broad range of aggregate formation (Figure 1).

### 3.2. Definition of Parameters Determining VHH Aggregation and Their Implementation to Calculate a Newly Introduced Aggregation Score

Intrigued by the observation that small changes such as the replacement of an alanine with a valine or the deletion/insertion of a single amino acid can have drastic effects on aggregation behavior, the deep learning model NanoBodyBuilder2 [36] was used to model the VHH structures aimed at identifying possible factors that may promote aggregation of VHHs. The structures predicted suggested altered hydrophobicity characteristics of the exposed FR2 surface, which is located below the CDR3 loop, as a possible aggregation determinant (Figure S1). Hence, we focused on the FR2 structural patch, defined as the region between sequence positions 39 and 55, according to IMGT numbering [34], also previously associated with stability and aggregation propensities [19].

**Figure 2:**
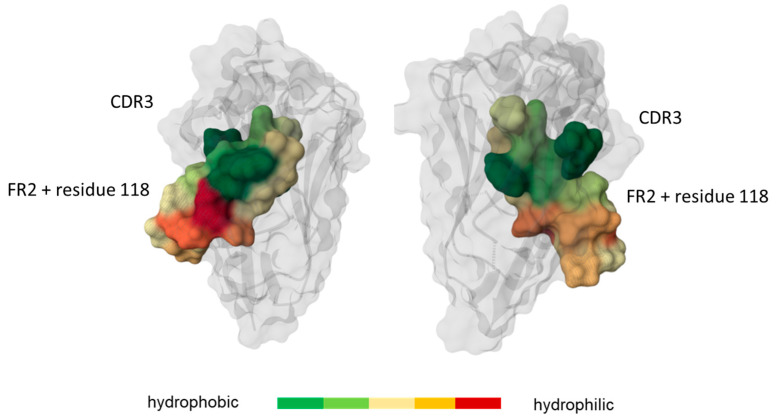
Graphical representation of the framework region 2 (FR2) and residue 118. FR2 is composed of residues 39 to 55, according to IMGT numbering. FR2 is structurally located below the CDR3 loop. The region is highlighted according to the hydrophobicity of its residues: green for hydrophobic ones and red for hydrophilic ones. A cartoon representation and a molecular surface representation were generated using Mol* [37].

Since hydrophobic solvent-accessible surface areas (SASs) significantly contribute to aggregation propensity, we estimate the overall aggregation tendency as the product of the exposed surface area and the corresponding amino acid hydrophobicity [40]. The hydrophobicity of these surfaces can be determined by the Wimley–White hydrophobicity scale [39], which quantifies the tendency of amino acid residues to associate with membrane interfacial regions. We also analyzed intramolecular interactions, since leucine, which was deleted in our humanized variant, seemed to have a stabilizing effect. We observed that the amount of intramolecular hydrophobic interactions, which facilitate stabilization of the hydrophobic core, was altered in aggregation-prone VHHs compared to variants with low aggregation propensity. The aggregation-prone clones VHH1 and VHH1_LAS had lower amounts of stabilizing intramolecular interactions in FR2, thereby leaving more conformational flexibility for the FR2 residues, rendering them more accessible for intermolecular interactions. In addition to FR2, the residue at position 118, according to IMGT numbering in FR4, involved in the VH:VL interface, was analyzed for its predicted structural and biophysical properties (Figure 2) [11,18].

Based on these analyses, we developed an aggregation score (AS) function (Formula (1)) that considers three VHH properties: (I) the hydrophobicity of the conserved immunoglobulin domain interaction interface (FR2 + residue118), (II) the mean hydrophobic intramolecular interactions possible for each residue in a radius of 4 Å of the contact interface (FR2 + residue118), and (III) the instability index of FR2. For calculation of term (I), the exposed surface area for each FR2 residue and residue 118 was multiplied with the hydropathy of the corresponding amino acid to calculate *AixHi*. Importantly, this term becomes smaller with the increased hydrophobicity of the surface area. By analyzing the intramolecular interaction (*hi*), summed up in term (II), information about the potential intermolecular hydrophobic interactions is obtained if we assume that the hydrophobic interaction potential is limited for each residue. Therefore, making intermolecular interactions less likely if the residue is incorporated in multiple intramolecular interactions. The higher the number of stabilizing interactions, the lower the potential of intermolecular interactions by the FR2 area, and the higher this term. The third term (III) used for calculating the AS is the instability index (i-i) [42]. This parameter was included since it was reported that the composition of dipeptide pairs within the primary structure of a given protein is significantly correlated with its stability. Based on this finding, Guruprasad and coworkers calculated a weight value of instability for each individual dipeptide. For the purpose of this work, and again focusing on the FR2 patch, the instability index was calculated for all dipeptides in the FR2 of a given VHH. A higher instability index indicates increased intrinsic instability; thus, the AS is proportional to the reciprocal of i-i. The absolute value of the score is used in the final calculation to improve the interpretability.

(1) $$\;AggregationScore\;(AS)=\left|\frac{\frac{1}{n}\sum_{i=1}^{n}\left(A_{i}\times H_{i}\right)\times \frac{1}{n}\sum_{i=1}^{n}h_{i}}{\frac{10}{L}\sum_{i=1}^{L-1}DIWV(x_{i},\;y_{i+1})}\right|$$

$$I=\frac{1}{n}\sum_{i=1}^{n}\left(A_{i}\times H_{i}\right)$$

$$II=\frac{1}{n}\sum_{i=1}^{n}h_{i}$$

$$III=\frac{10}{L}\sum_{i=1}^{L-1}DIWV(x_{i},\;y_{i+1})$$

*Ai*: numerical value of the exposed surface area of residue I;
*Hi*: hydrophobicity of residue I;
*hi*: hydrophobic intramolecular interactions of residue I;
*DIWV*: dipeptide instability weight value.

### 3.3. The VHH Interface FR2 as an Aggregation Determinant for Recombinant Nanobody Aggregation

**Figure 3:**
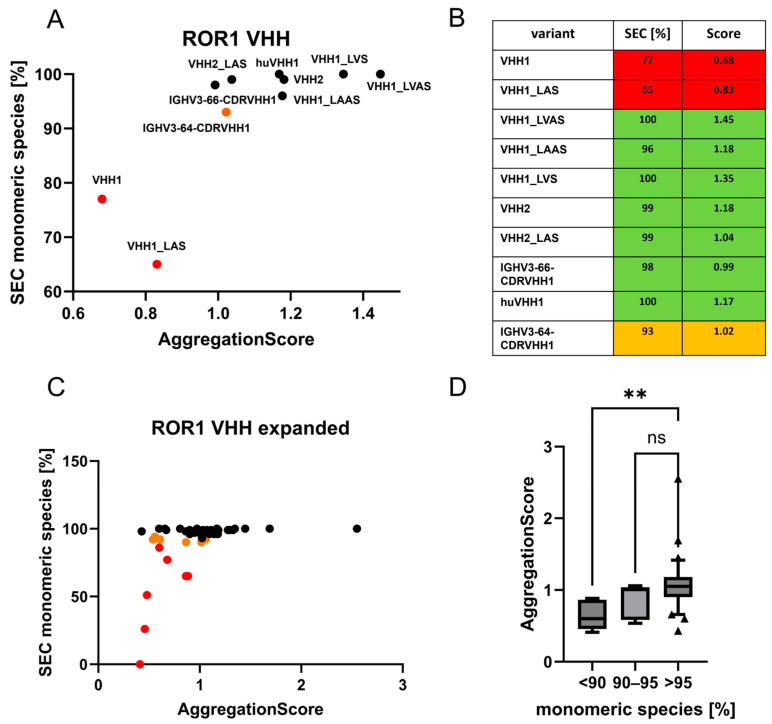
Aggregation propensities of ROR1-targeting VHHs in relation to their predicted score obtained by the employment of Formula 1. (**A**) Plot showing the monomeric species in SEC on the y-axis and the calculated aggregation score on the x-axis. The set consists of camelid VHH1 and VHH2 and humanized clones of those. VHHs with less than 90% of monomeric species are highlighted in red, with values between 90 and 95% in orange, and above 95% in black. VHHs showing more than 5% aggregation have a score below 1. (**B**) List of ROR1 VHHs with their measured monomeric species in SEC and their calculated aggregation score. VHHs with more than 95% monomeric species are highlighted in green, between 90 and 95% in orange, and below 90% in red. (**C**) Plot showing the monomeric species in SEC on the x-axis and the calculated score on the y-axis. The set consists of 48 αROR1 VHHs. VHHs with less than 90% monomeric species are highlighted in red, with between 90 and 95% in orange, and above 95% in black. (**D**) Box plot of all 48 ROR1-targeting VHHs, with whiskers indicating the 10th to 90th percentiles. VHHs are grouped according to their monomeric species content in SEC: <90% (n = 7); 90–95% (n = 9); >95% (n = 32). The significance was analyzed using a Kruskal–Wallis test (** *p* ≤ 0.01; ns *p* > 0.05).

We determined the aggregation score of the VHH2- and VHH1-derived VHH variants to see if these correlate with the observed aggregation in SEC. For the aggregating VHHs, VHH1 and VHH1_LAS, we observed lower ASs compared to the non-aggregating VHHs (Figure 3A,B). VHHs with more than 95% monomeric species in SEC had a score between 0.99 and 1.45, with an average score of 1.19. The VHH IGHV3-66-CDRVHH1, which had 93% of monomer species in SEC, had a score of 1.02. The highly aggregating VHHs VHH1 and VHH1_LAS had scores of 0.68 and 0.83, respectively, resulting in an average score of 0.78. All VHHs with a score higher than 1 showed more than 90% of monomer species, whereas the two aggregating VHHs had a score lower than 0.85. These results open up the possibility of adapting AS thresholds to the requirements of a given selection process for promising VHHs.

Motivated by this finding, we included 38 more ROR1-targeting VHHs, 48 in total, from our clone collection, for which previously obtained aggregation data from SEC analysis were available (Figure 3C). We observed an average score of 1.08 for the 32 VHHs with more than 95% of monomeric species in SEC, an average score of 0.86 for the 9 VHHs with monomeric species between 90 and 95%, and a score of 0.62 for the VHHs with less than 90% of monomeric species. This resulted in a significant difference in VHHs with more than 95% of monomeric species and those with less than 90% monomeric species (Figure 3D). For example, a separation threshold of 1 can correctly classify 19 of the 32 VHHs with more than 95% of monomeric species, 5 of the 9 VHHs with 90–95% of monomeric species, and none of the VHHs with less than 90% of monomeric species.

### 3.4. The Aggregation Score as a Tool to Predict Aggregation Propensities in a Recombinant VHH Collection-Targeting Antigen 2

**Figure 4:**
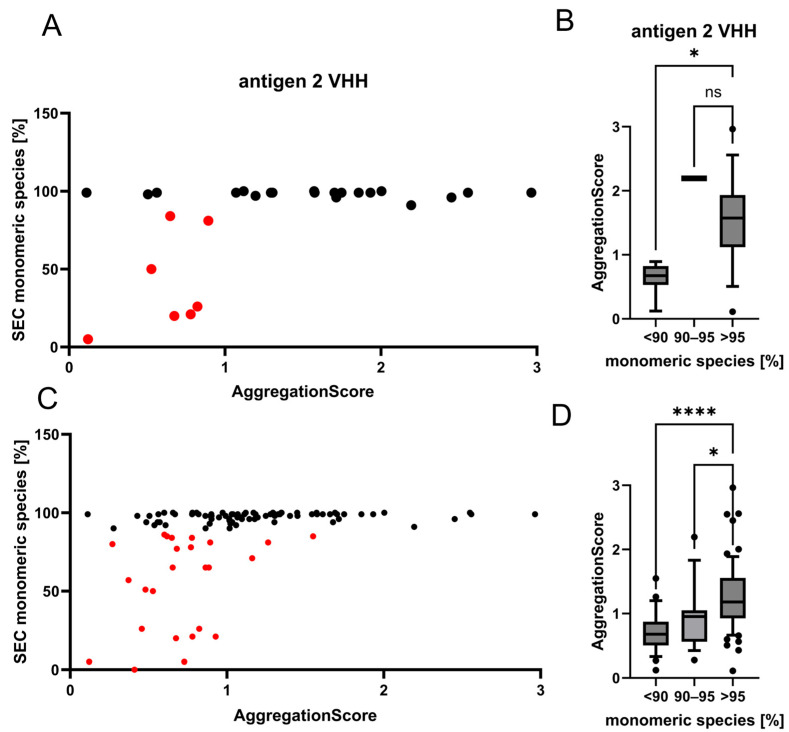
(**A**) Aggregation score related to monomer content for a collection of antigen 2-targeting VHHs. Plot showing the monomeric species in SEC on the x-axis and the calculated score on the y-axis. The set consists of 27 antigen 2 VHHs. VHHs with less than 90% of monomeric species are highlighted in red and above 90% in black. (**B**) Box plot of all 27 antigen 2-targeting VHHs, with whiskers indicating the 10th to 90th percentiles. VHHs are grouped according to their monomeric species content in SEC: <90% (n = 7); 90–95% (n = 1); >95% (n = 19). Significance was analyzed using the Kruskal–Wallis test (* *p* ≤ 0.05; ns *p* > 0.05). (**C**) Overview of the score of all 106 VHHs analyzed in this work in correlation to the measured monomeric content in SEC. (**D**) Box plot of 106 VHHs with whiskers showing 10–90% percentile. VHHs are grouped according to their monomeric species content in SEC: <90% (n = 25); 90–95% (n = 16); >95% (n = 65). The significance was analyzed using the Kruskal–Wallis test (**** *p* ≤ 0.0001; * *p* ≤ 0.05).

To exclude a possible bias resulting from the consideration of VHHs directed against a particular target, we wanted to evaluate the score function for another screening project, which delivered VHHs directed against an unrelated target. The VHHs were obtained from a different camelid immunization (Table S2). In this project, 15 VHHs were isolated, and 2 of those clones (1072 and c4) were humanized, resulting in 12 humanized variants differing in their amino acids at the Vernier residues of the IGVH3-23 framework. Out of these 27 variants, 19 had more than 95% of monomeric species in SEC, with an average AS of 1.54. VHH 1063 with 91% monomeric species in SEC had an AS of 2.19, and seven VHHs with less than 90% had AS between 0.12 and 0.89, with an average of 0.64. This resulted in a significant difference between those with more than 95% and those with less than 90% (Figure 4A,B). Application of a threshold of 1 resulted in the coverage of 16 out of 19 VHHs with more than 95% and the inclusion of no VHH with less than 90% of monomeric species.

To further evaluate if this method is generally suitable to categorize VHHs with different aggregation propensities, the aggregation score was tested for its predictive performance on 106 different VHHs in total, including both the above-introduced collections. These 106 VHHs were obtained from 4 different camelid immunizations (3 Bactrian camels and 1 alpaca) as well as from synthetic libraries. A total of 65 VHHs had more than 95% of monomeric species in SEC, with an average AS of 1.26. A total of 16 VHHs had between 90 and 95% of monomeric species and an average AS of 0.94. A total of 25 VHHs had less than 90% of monomeric species in SEC, with an average AS of 0.71. The difference between the VHHs with more than 95% and the other groups is significant (Figure 4C,D). By applying a threshold of 1, 46 out of 65 VHHs with more than 95% of monomeric species, 8 out of 16 VHHs with 90–95%, and 3 out of 25 VHHs with less than 90% of monomeric species would be selected. This would result in an 88% reduction of highly aggregating VHHs in the selected set, while retaining the vast majority of non-aggregating VHHs for further studies. Additionally, we conducted a more comprehensive analysis of potential thresholds (Figures S3–S5 and Tables S3 and S4). Depending on the dataset size and the number of clones to select, either precision- or recall-focused thresholds can be applied. Using a threshold of >0.89, corresponding to the Youden index J and F0.5max, 61 out of 81 VHHs with ≥90% of monomeric species and 4 out of 25 aggregation-prone VHHs would be obtained, yielding an accuracy of 0.77 and a balanced accuracy of 0.80. A lower threshold of >0.48, corresponding to F1max, would obtain 78 out of 81 non-aggregation-prone VHHs but also 19 out of 25 aggregation-prone VHHs, resulting in an accuracy of 0.79 and a balanced accuracy of 0.60. For VHHs with ≥95% of monomeric species, a threshold of >1.06, which corresponds to F0.5max, would obtain 43 out of 65 non-aggregation-prone VHHs and 5 out of 41 aggregation-prone VHHs, corresponding to an accuracy of 0.75 and a balanced accuracy of 0.76. These results indicate that higher thresholds favor precision by reducing aggregation-prone VHHs, whereas lower thresholds favor recall by including more non-aggregation-prone VHHs, albeit with more false positives.

### 3.5. FR2 Sequence Cluster Analysis in Correlation with VHH Aggregation Propensity

**Figure 5:**
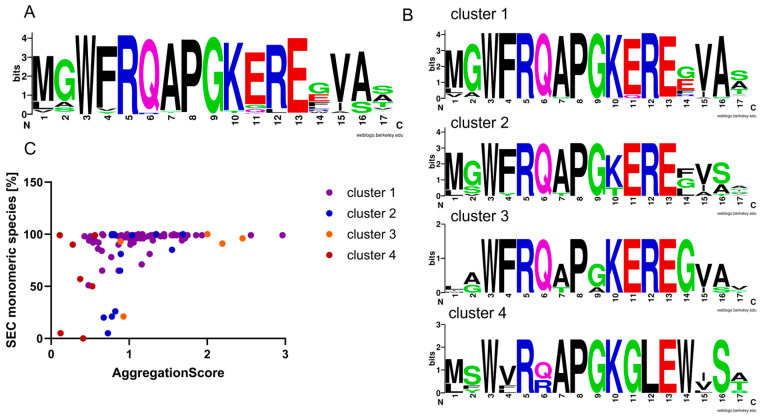
Clustering of VHHs according to their FR2 sequence composition. (**A**) FR2 sequence logo from all 106 VHHs. Amino acids are colored according to their chemical properties: small polar amino acids (G, S, T, C, Y) are shown in green; amide-containing polar amino acids (Q, N) are shown in purple; basic amino acids (K, R, H) are shown in blue; acidic amino acids (D, E) are shown in red; and hydrophobic amino acids (A, V, L, I, P, W, F, M) are shown in black [44,45]. (**B**) Sequence logos of four FR2 clusters derived from groups comprising at least four individual VHHs obtained by MMseqs [41,42]. Cluster 1 consists of 69 VHHs, cluster 2 of 14 VHHs, cluster 3 of 5 VHHs, and cluster 4 of 7 VHHs. (**C**) Plot showing the monomeric species in SEC on the x-axis and the calculated score on the y-axis. VHHs of cluster 1 are highlighted in purple, cluster 2 in blue, cluster 3 in orange, and cluster 4 in red.

To investigate the influence of the FR2 primary sequence composition on the predicted aggregation propensities, a cluster analysis for the FR2 sequences was performed. For this purpose, MMseqs2 was used with a minimum sequence identity of 0.8 and a minimum alignment coverage of 0.9 [41,42,43]. In total, four distinguishable clusters were identified (Figure 5). VHHs harboring three of these FR2 sequence clusters deliver calculated aggregation scores, which allow for the distinction between variants with high versus low aggregation propensity. In contrast, cluster 4, which was characteristic for VHHs harboring the canonical VH-associated residues “VGLW”, was identified as the cluster with the lowest aggregation score on average but contained variants with low aggregation behavior (Figure 5C and Figure S2). The reason for this unexpected discrepancy is currently under investigation.

### 3.6. Superiority of the FR2-Restricted Aggregation Score for Nanobodies over Aggregation Prediction Considering the Entire Protein

To further verify the predictive potential of the FR2 patch of nanobodies analyzed by the presented aggregation score, we compared its performance with that of AGGRESCAN3D 2.0 [21].

Intergroup variance between the score values of the 106 VHHs with ≤90, 90–95, and ≥95% of monomeric species was assessed but did not demonstrate significant heterogeneity, as indicated by the Levene test (*p* = 0.153). Consequently, the Kruskal–Wallis test was employed for intergroup comparison, as the distribution within the “Over 95%” group deviated from normal distribution. Dunn’s multiple comparison test was applied, allowing for statistically robust pairwise evaluation of group-wise differences.

The FR2-based aggregation score yielded a highly significant distinction between VHHs showing ≤ 90% and ≥95% of monomeric species (Figure 3 and Figure 4). In contrast, the analogous comparison using AGGRESCAN3D 2.0 showed no significance (Figure S6). The difference between the 90–95% and ≥95% groups, although small, remained statistically significant applying the aggregation score presented in this work. Additionally, a more rigorous statistical comparison was performed, including receiver operating characteristic (ROC) curves, precision–recall curves, confusion matrices, and calculations of accuracy and balanced accuracy at different thresholds, based on the Youden index J and various Fβmax values (Figures S3–S5 and Tables S3 and S4). Overall, the aggregation score showed higher accuracies and balanced accuracies, particularly for distinguishing VHHs with at least 95% of monomeric species.

Aggregation predictions via AGGRESCAN3D 2.0 showed that comparing the score of whole VHH structures with and without inclusion of the FR2 regions (residues 39–55) presents an average score shift of 7.8% (Figure S7). This minimal deviation suggests that AGGRESCAN3D 2.0 lacks sensitivity to the key contributions of the FR2 region and may not effectively resolve its impact on aggregation propensity.

### 3.7. Application of the Aggregation Score to Predict Properties of Synthetic VHHs Based on the Identical Framework

**Figure 6:**
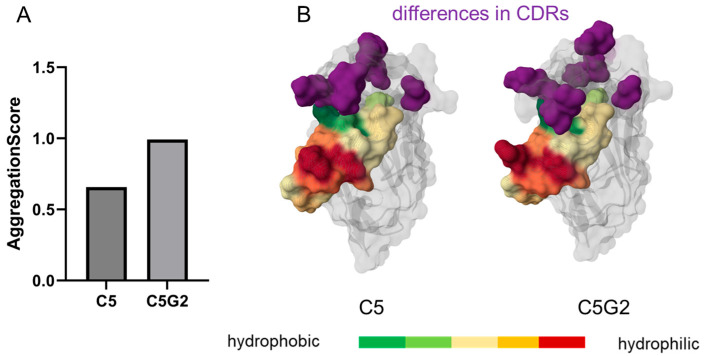
Comparison of two synthetic clones, C5 and C5G2, comprising the identical framework. (**A**) AS for the synthetic clone C5, which showed aggregation, and the affinity-matured clone C5G2, which showed no aggregation. (**B**) Predicted structures for the clones C5 and C5G2. For FR2 and residue 118, the hydrophobic residues are colored green, and the hydrophilic ones in red. Changes in the CDR regions are highlighted in purple.

Our findings suggest that the aggregation score is also applicable to compare VHHs comprising an identical framework but different CDRs. For instance, the studied VHHs VHH1_LAS and VHH2_LAS show notable differences in their aggregation behavior. Next, we wanted to determine if the AS might also be applicable to synthetic libraries using a fixed framework. For this purpose, we chose nanobody C5, targeting the RBD domain of the spike protein of SARS-CoV-2 [46]. This VHH was obtained from a synthetic library and showed aggregation and poor expression. After affinity maturation by CDR randomization, a clone with improved affinity and expression properties, termed C5G2, was obtained [46]. C5G2 has the same framework as C5 and differs in the CDR regions. This clone showed no aggregation in SEC. We compared the scores for the two VHHs and observed that C5 had an AS of 0.66 and C5G2 of 0.99. By comparing the predicted structures, we noted that the mutations in the CDRs affect the shielding of the hydrophobic residues in FR2 and in position 118 (Figure 6). The CDR3 loop is predicted to more intensely interfere with the aggregation determining the FR2 area and, thereby, prevent aggregation. This finding suggests that the AS presented in this work is also applicable for synthetic libraries using a fixed framework.

## 4. Discussion

Aggregation of proteins is a complex process influenced by various factors. We investigated which parameters are suitable to predict aggregation of VHHs based on their sequence.

Most notably, in contrast to previously published tools, such as AGGRESCAN3D 2.0, which are based on entire protein 3D structure predictions, our approach highlights the relevance of the FR2 region as a meaningful proxy for aggregation prediction for VHH domains. Moreover, unlike AGGRESCAN3D 2.0, the aggregation score function introduced in this work includes intramolecular interactions within the FR2. For these reasons, the aggregation score is an efficient and rational device to identify aggregation-prone nanobody candidates during the early discovery and development process.

CDR3 length, CDR3 charge, and the presence of an additional disulfide bridge have been reported to have an impact on the aggregation of VHHs [19,47,48,49,50,51]. Notably, no correlation between CDR3 length and aggregation was observed for the set of VHHs analyzed in this work (Figure S8). VHHs with additional disulfide bridges showed less aggregation as well as camelid VHHs. This is in line with reports from the literature showing that the additional non-canonical disulfide bridge can stabilize the VHH [16]. However, these parameters do not allow us to distinguish between aggregation-prone and stable VHHs in an efficient manner. Therefore, we utilized deep learning-based structure predictions to gain insight into putative biophysical properties, since we assumed that CDRs, particularly long CD3 loops, could contribute to shielding hydrophobic patches in the FR2 region from aggregate formation. We focused on the consensus intermolecular immunoglobulin interaction interface FR2 and developed a scoring function based on accessibility and shielding of this surface region. By considering hydrophobicity, hydrophobic intramolecular interactions, and an empirical instability index based on the dipeptide composition, we devised a score function that was able to distinguish between VHHs with high and low aggregation propensity. In contrast, a multi-linear analysis of primary sequence-based parameters of entire proteins did not yield a comparable distinction.

By analyzing FR2 sequence clusters for their relevance in this context, we noticed that VHHs with the VGLW motif are an exception, since their aggregation behavior cannot be predicted by the presented function. For all members of this group, a relatively low aggregation score is obtained, which does not correlate with their actual aggregation behavior. Therefore, VHHs belonging to that FR2 cluster should not be evaluated for aggregation propensity with this model and, instead, should be analyzed experimentally. The reason for the low correlation of aggregation score and actual aggregation behavior is presently unclear and warrants further experimental validation, since our available dataset for VHHs of this cluster, seven in total, is restricted. Structural methods, such as X-ray crystallography, and biophysical approaches, such as nano differential scanning fluorimetry (nanoDSF), as well as computational techniques, like molecular dynamics (MD) simulations or AlphaFold Multimer modeling of multiple VHHs, could provide new insights into this.

Furthermore, a small number of VHHs from other clusters are predicted to be aggregation-prone and, despite that, showed low amounts of aggregation in the SEC analysis. Possible reasons might be that the shielding by the CDR3 loop is not accurately reflected in the predicted structure or that there are other stabilizing factors, which are not considered in this model. VHHs harboring an additional disulfide bond tended to be less aggregation-prone (Figure S8B), consistent with previous reports indicating that the non-canonical disulfide bridge provides additional stabilization [16]. Incorporating a bonus in the aggregation score calculation for the presence of an extra disulfide bridge could be considered for future predictive models. Further improvements to the model could be achieved by integrating additional sequence-based properties of the CDR loops—particularly the CDR3 loop—such as length, hydrophobicity, and charge distribution (e.g., charge patches) based on amino acid composition. For example, we observed that some VHHs with high aggregation scores but substantial aggregation had particularly hydrophobic CDRs. Given the challenges in accurately predicting the structure of CDR loops, especially the long and diverse loops typical of VHHs, achieving high accuracy using structural features alone may be difficult. However, incorporating compositional and physicochemical properties offers a strong potential to improve the model’s ability to distinguish between aggregation-prone and stable VHHs and represents a valuable direction for future development. For instance, VHHs 294 and 248 had scores of 1.16 and 1.55, respectively, but only 71% and 85% of monomeric species, as measured by SEC. A sequence analysis of the CDR loops revealed that 248 has a relatively short, yet quite hydrophobic, CDR3 loop, while 294 features a notably hydrophobic CDR2 loop. Short CDR loops might not provide sufficient shielding of aggregation-prone regions, and hydrophobic patches within CDRs may further promote aggregation.

Notably, all 106 VHHs included in this study stem from animal immunization or humanization of those VHHs, where some “quality control” occurs during their generation in B cells, ensuring the selection of stable antibodies [52,53]. An alternative strategy for (humanized) nanobody discovery relies on the generation of synthetic libraries with randomized CDR sequences that are eventually screened by phage display or ribosomal display [54,55]. We analyzed two synthetic clones for which the AS was markedly lower in the case of the aggregation-prone VHH. However, a larger dataset needs to be tested to evaluate whether our prediction tool for aggregation behavior is also applicable to this class of VHHs. Furthermore, aggregation was only investigated by SEC. Therefore, additional orthogonal methods, such as dynamic light scattering (DLS), could be valuable for cross-validation. Hydrophobic interaction chromatography, kinetic measurements of VHH aggregation, and differential melting temperature analyses, as described in Kunz et al., might also be of interest [19]. Nevertheless, we show that for VHHs obtained from animal immunization that contain camelid or human framework sequences, the aggregation score can be utilized in a screening project to eliminate potentially aggregation-prone VHHs, therefore reducing time and resources in the early discovery and development process.

A central task for future work is to enhance the precision and accuracy of aggregation predictions by the integration of further knowledge of key VHH domain dynamics based on molecular dynamics modeling. To this end, targeted molecular dynamic simulations, in-depth data mining of the existing data, and the evaluation of various neuro-symbolic AI models for improved classification are planned using the xyna.bio platform. The expansion of the database with additional experimental and synthetic data is essential. Equally important is the inclusion of de novo in silico nanobodies, which can be developed using AI-based methods. These potential synthetic frameworks could then be systematically evaluated. The current AS model is now freely available as a web server application on the xyna.bio platform.

## Supplementary Materials

The following supporting information can be downloaded at https://www.mdpi.com/article/10.3390/antib14030073/s1, Figure S1: Structural investigation of the VHH region analog to the VH VL interface for different ROR1-targeting VHHs. Hydrophobic residues are highlighted in green, and hydrophilic ones in red. VHH1_LAS and parental VHH1 were aggregation-prone. Both VHHs have an exposed hydrophobic former VL interface. In contrast, this region is more shielded for the VHH1_LVAS variant, which did not aggregate. Figure S2: Overview of different FR2 clusters. A. Plot showing the monomeric species in SEC on the x-axis and the calculated score on the y-axis for cluster 1 (n = 33). B. Plot showing the monomeric species in SEC on the x-axis and the calculated score on the y-axis for cluster 2 (n = 14). C. Plot showing the monomeric species in SEC on the x-axis and the calculated score on the y-axis for cluster 3 (n = 5). D. Plot showing the monomeric species in SEC on the x-axis and the calculated score on the y-axis for cluster 4 (n = 7). Figure S3: Receiver operating characteristic (ROC) curve for aggregation score and AggreScan3D. Separation into VHHs with ≥90% of monomeric species in SEC resulted in 81 positive VHHs (*p* = 81) and 25 negative VHHs (N = 25). Separation into VHHs with ≥95% of monomeric species in SEC resulted in 65 positive VHHs (*p* = 65) and 41 negative VHHs (N = 41). A. ROC curve of the aggregation score for separation of VHHs with ≥90% of monomeric species in SEC. An area under the ROC curve (AUROC) of 0.81 was obtained with *p* < 0.0001. A Youden index J of 0.59 was obtained. B. ROC curve of aggregation score for separation of VHHs with ≥ 95% of monomeric species in SEC. An AUROC of 0.79 was obtained with *p* < 0.0001. A Youden index J of 0.52 was obtained. C. ROC curve of AggreScan3D for the separation of VHHs with ≥ 90% of monomeric species in SEC. An AUROC of 0.65 was obtained with *p* = 0.0223. A Youden index J of 0.33 was obtained. D. ROC curve of AggreScan3D for the separation of VHHs with ≥ 95% of monomeric species in SEC. An AUROC of 0.53 was obtained with *p* = 0.6015. A Youden index J of 0.19 was obtained. Figure S4: Precision–recall curves (PRC) for aggregation score and AggreScan3D. Separation into VHHs with ≥90% of monomeric species in SEC resulted in 81 positive VHHs (*p* = 81) and 25 negative VHHs (N = 25). Separation into VHHs with ≥95% of monomeric species in SEC resulted in 65 positive VHHs (*p* = 65) and 41 negative VHHs (N = 41). A. PRC of the aggregation score for separation of VHHs with ≥ 90% of monomeric species in SEC. An area under the precision–recall curve (AUPRC) of 0.93 and an F1max of 0.88 was obtained. B. PRC of the aggregation score for separation of VHHs with ≥ 95% of monomeric species in SEC. An area under the precision–recall curve (AUPRC) of 0.84 and an F1max of 0.82 was obtained. C. PRC of the AggreScan3D for separation of VHHs with ≥90% of monomeric species in SEC. An area under the precision–recall curve (AUPRC) of 0.84 and an F1max of 0.88 was obtained. D. PRC of the AggreScan3D for separation of VHHs with ≥95% of monomeric species in SEC. An area under the precision–recall curve (AUPRC) of 0.63 and an F1max of 0.77 was obtained. Figure S5: Confusion matrix for different thresholds for aggregation score and AggreScan3D. These thresholds were chosen based on the Youden index (J) from the receiver operating characteristic (ROC) curve and the maximum F1, F0.5, and F2 values from the precision–recall curve. Different β values were applied when calculating the maximum F value to adjust the focus toward the recall sensitivity or precision sensitivity: β > 1 emphasizes recall, while β < 1 emphasizes precision. For a recall-oriented evaluation, a β value of 2 was selected; for a precision-oriented evaluation, a β value of 0.5 was selected. For equal weighting of precision and recall, a β value of 1 was selected. In Q1, the number of true positives (TPs) is displayed; in Q2, false negatives (FNs); in Q3, false positives (FPs); and in Q4, true negatives (TNs). The matrices are color-coded, with green representing the optimal value (TP = P; TN = N; FP = 0; FN = 0). A. Confusion matrices for the aggregation score for separating VHHs with ≥ 90% of monomeric species in SEC (*p* = 81; N = 25). By applying the threshold for the Youden index J (>0.89), an accuracy of 0.77 and a balanced accuracy of 0.8 were obtained. For the corresponding thresholds, for F1max, an accuracy of 0.79 and a balanced accuracy of 0.6; for F0.5max, an accuracy of 0.77 and a balanced accuracy of 0.8; and for F2max, an accuracy of 0.77 and a balanced accuracy of 0.53 were obtained. B. Confusion matrices for the AggreScan3D for separating VHHs with ≥90% of monomeric species in SEC (*p* = 81; N = 25). By applying the threshold for the Youden index J (≤−91), an accuracy of 0.76 and a balanced accuracy of 0.66 were obtained. For the corresponding thresholds for F1max, an accuracy of 0.76 and a balanced accuracy of 0.52; for F0.5max, an accuracy of 0.73 and a balanced accuracy of 0.67; and for F2max, an accuracy of 0.76 and a balanced accuracy of 0.52 were obtained. C. Confusion matrices for the aggregation score for separating VHHs with ≥95% of monomeric species in SEC (*p* = 65; N = 41). By applying the threshold for the Youden index J (>0.89), an accuracy of 0.77 and a balanced accuracy of 0.75 were obtained. For the corresponding thresholds, for F1max, an accuracy of 0.75 and a balanced accuracy of 0.71; for F0.5max, an accuracy of 0.75 and a balanced accuracy of 0.76; and for F2max, an accuracy of 0.65 and a balanced accuracy of 0.53 were obtained. D. Confusion matrices for the AggreScan3D for separating VHHs with ≥95% of monomeric species in SEC (*p* = 65; N = 41). By applying the threshold for the Youden index J (≤−91), an accuracy of 0.65 and a balanced accuracy of 0.59 were obtained. For the corresponding thresholds, for F1max, an accuracy of 0.62 and a balanced accuracy of 0.51; for F0.5max, an accuracy of 0.65 and a balanced accuracy of 0.59; and for F2max, an accuracy of 0.62 and a balanced accuracy of 0.51 were obtained. Figure S6: Analysis of the whole VHH dataset by AggreScan3D 2.0. A. Plot showing the total score on the x-axis and the monomeric species in SEC on the y-axis. B. Box plot of 106 VHHs with Whisker showing the 10–90% percentile. VHHs are grouped according to their monomeric species content in SEC: <90% (n = 25); 90–95% (n = 16); >95% (n = 65). The significance was analyzed using a one-way Kruskal–Wallis test (**** *p* ≤ 0.0001; *** *p* ≤ 0.001; ** *p* ≤ 0.01; *p* ≤ 0.05; ns *p* > 0.05). Figure S7: Relevance of the FR2 sequence patch for the prediction of VHH aggregation by AGGRESCAN3D 2.0. Aggregation behavior of 106 full-length VHH sequences, correlated with aggregation propensities predicted by AGGRESCAN3D 2.0, with (black) and without (green) the FR2 region (residues 39–55), normalized using min–max normalization. Thresholds for 90% (light red) and 95% (red) monomeric species, as determined by size exclusion chromatography (SEC), are indicated. Exclusion of the FR2 region results in an average aggregation score shift of 7.8%. Figure S8: Impact of CDR3 length, the presence of non-canonical disulfide bridge, and humanization on aggregation behavior of VHHs. A. Plot showing the monomeric species in SEC on the y-axis and the CDR3 length on the x-axis. No significant correlation was observed with a Pearson coefficient of 0.14. B. Scatter plot comparing the aggregation for VHHs with and without non-canonical disulfide bridges. A total of 54 VHHs had an additional disulfide bridge and an average of 93.4% monomeric species in SEC, whereas 52 VHHs had no additional disulfide bridge and had on average 81.1% monomeric species in SEC. C. Scatter plot comparing the aggregation for VHHs that are humanized or camelid. A total of 36 VHHs were humanized and showed on average 79.9% monomeric species in SEC, whereas 70 VHHs were not humanized and showed on average 91.2% monomeric species in SEC. The significance was analyzed using an unpaired *t*-test (*** *p* ≤ 0.001; ** *p* ≤ 0.01; *p* ≤ 0.05; ns *p* > 0.05). Table S1: Origin, monomeric species measured in SEC, and calculated score for ROR1-targeting VHHs. Table S2: Origin, monomeric species measured by SEC, and calculated score for VHH-targeting antigen 2. Table S3: Summary of statistical metrics for separating VHHs with ≥90% of monomeric species in SEC (*p* = 81; N = 25) comparing aggregation score and AggreScan3D. Table S4: Summary of statistical metrics for separating VHHs with ≥95% of monomeric species in SEC (*p* = 65; N = 41) comparing aggregation score and AggreScan3D.

## Abbreviations

The following abbreviations are used in this manuscript:

**Abbreviation**	**Full Term**
**A3D**	AGGRESCAN3D 2.0
**AI**	Artificial Intelligence
**Ai**	Accessible Surface Area of Residue i
**AS**	Aggregation Score
**ASA**	Accessible Surface Area
**BLI**	Bio-Layer Interferometry
**cDNA**	Complementary DNA
**CDR**	Complementarity-Determining Region
**DNA**	Deoxyribonucleic Acid
***E. coli***	*Escherichia coli*
**FACS**	Fluorescence-Activated Cell Sorting
**Fc**	Fragment Crystallizable Region
**FN**	False Negative
**FP**	False Positive
**FR**	Framework Region
**FR2**	Framework Region 2
**FR4**	Framework Region 4
**GL column**	Gel Filtration Column (Superdex 200 Increase 10/300 GL)
**Hi**	Hydropathy Index of Residue i
**hi**	Hydrophobic Interactions of Residue i
**Ig**	Immunoglobulin
**IgBLAST**	Immunoglobulin Basic Local Alignment Search Tool
**IGHV**	Immunoglobulin Heavy Variable
**i-i**	Instability Index
**IMGT**	International ImMunoGeneTics Information System
**IMGT numbering**	International ImMunoGeneTics Information System Numbering Scheme
**nanoDSF**	Nano Differential Scanning Fluorimetry
**OPM medium**	Optimized Protein Medium
**PCR**	Polymerase Chain Reaction
**PDB**	Protein Data Bank
**ROR1**	Receptor Tyrosine Kinase-like Orphan Receptor 1
**SEC**	Size Exclusion Chromatography
**TN**	True Negative
**TP**	True Positive
**VH**	Variable Domain of Heavy Chain
**VH:VL**	Variable Heavy Chain to Variable Light Chain Interface
**VHH**	Variable Domain of Heavy Chain of Heavy-Chain-Only Antibodies (Nanobody)
**VL**	Variable Domain of Light Chain
**YSD**	Yeast Surface Display
