# Supplementary material for: Computational Prediction of Single-Domain Immunoglobulin Aggregation Propensities Facilitates Discovery and Humanization of Recombinant Nanobodies
Source: Antibodies (Basel). 2025 Aug 28;14(3):73. doi: 10.3390/antib14030073 (PMC12452744; doi:10.3390/antib14030073)
Supplement: Supplementary file 1 [file antibodies-14-00073-s001.zip › antibodies-3782977-supplementary.pdf]

antibodies

Article

Computational Prediction of Single Domain Immunoglobulin Aggregation Propensities Facilitates Discovery and Humanization of Recombinant Nanobodies

Felix Klaus Geyer [1], Julian Borbeck [2], Wiktoria Palka [2], Xueyuan Zhou [3], Jeffrey Takimoto [3], Brian Rabinovich[3], Bernd Reifenhäuser [2], Karlheinz Friedrich [2] and Harald Kolmar [1,4]*

[1] Clemens-Schöpf-Institut für Organische Chemie und Biochemie, 64287 Darmstadt, Germany;
[2] GIP AG, xyna.bio, 55131 Mainz, Germany
[3] Drug Discovery and Development, Fuse Biotherapeutics, Woburn, MA 01801, USA
[4] Centre for Synthetic Biology, Technical University of Darmstadt, 64283 Darmstadt, Germany
* Correspondence: harald.kolmar@tu-darmstadt.de

Supplementary Materials

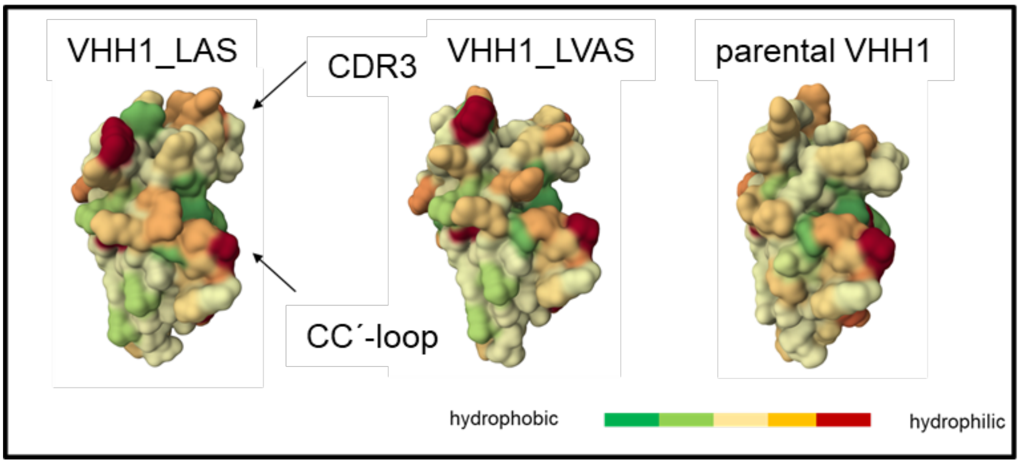

Figure S1. Structural investigation of the VHH region analog to VH VL interface for different ROR1 targeting VHHs. Hydrophobic residues are highlighted in green and hydrophilic ones in red. VHH1_LAS and parental VHH1 were aggregation prone. Both VHHs have an exposed hydrophobic former VL interface. In contrast this region is more shielded for the VHH1_LVAS variant, which did not aggregate.

 https://doi.org/10.3390/antib14030073

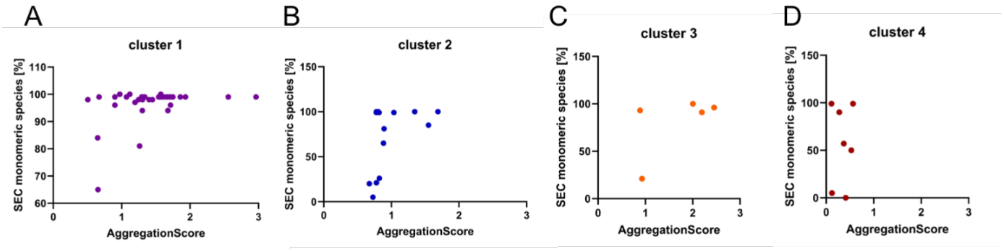

Figure S2. Overview of different FR2 clusters. **A** Plot showing the monomeric species in SEC on the x-axis and the calculated score on the y-axis for cluster 1 (n=33). **B** Plot showing the monomeric species in SEC on the x-axis and the calculated score on the y-axis for cluster 2 (n=14). **C** Plot showing the monomeric species in SEC on the x-axis and the calculated score on the y-axis for cluster 3 (n=5). **D** Plot showing the monomeric species in SEC on the x-axis and the calculated score on the y-axis for cluster 4 (n=7).

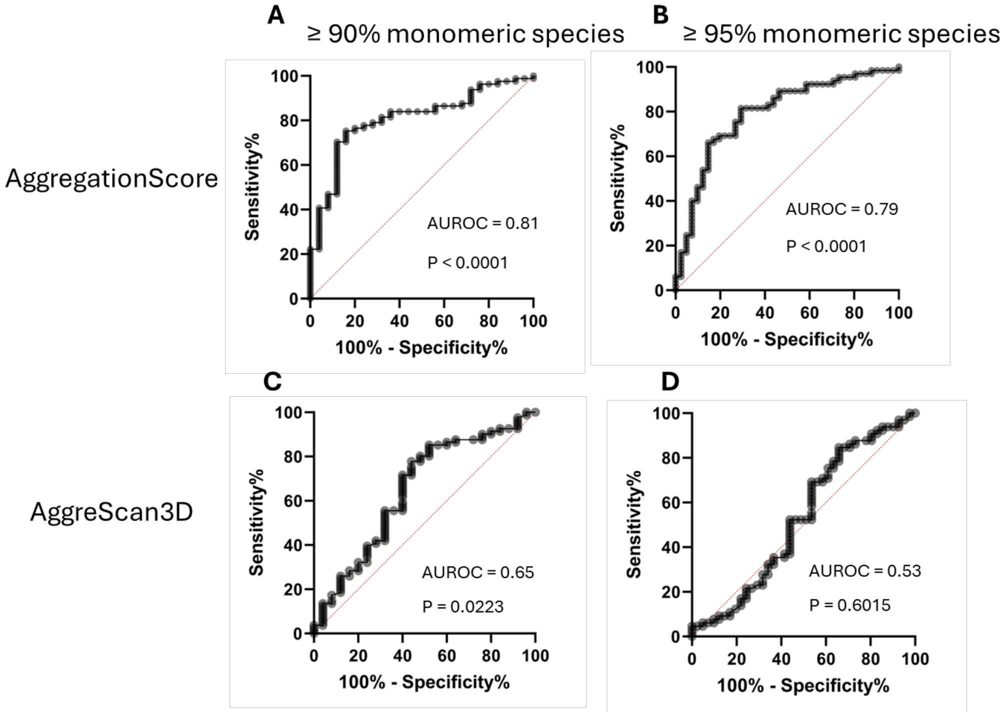

Figure S3: Receiver Operating Characteristic (ROC) curve for AggregationScore and AggreScan3D. Separation into VHHs with ≥ 90% monomeric species in SEC resulted in 81 positive VHHs (P=81) and 25 negative VHHs (N=25). Separation into VHHs with ≥ 95% monomeric species in SEC resulted in 65 positive VHHs (P=65) and 41 negative VHHs (N=41). **A** ROC curve of AggregationScore for separation of VHHs with ≥ 90% monomeric species in SEC. An area under the ROC curve (AUROC) of 0.81 was obtained with P<0.0001. A Youden Index J of 0.59 was obtained. **B** ROC curve of AggregationScore for separation of VHHs with ≥ 95% monomeric species in SEC. An AUROC of 0.79 was obtained with P<0.0001. A Youden Index J of 0.52 was obtained. **C** ROC curve of AggreScan3D for separation of VHHs with ≥ 90% monomeric species in SEC. An AUROC of 0.65 was obtained with P=0.0223. A Youden Index J of 0.33 was obtained. **D** ROC curve of AggreScan3D for separation of VHHs with ≥ 95% monomeric species in SEC. An AUROC of 0.53 was obtained with P=0.6015. A Youden Index J of 0.19 was obtained.

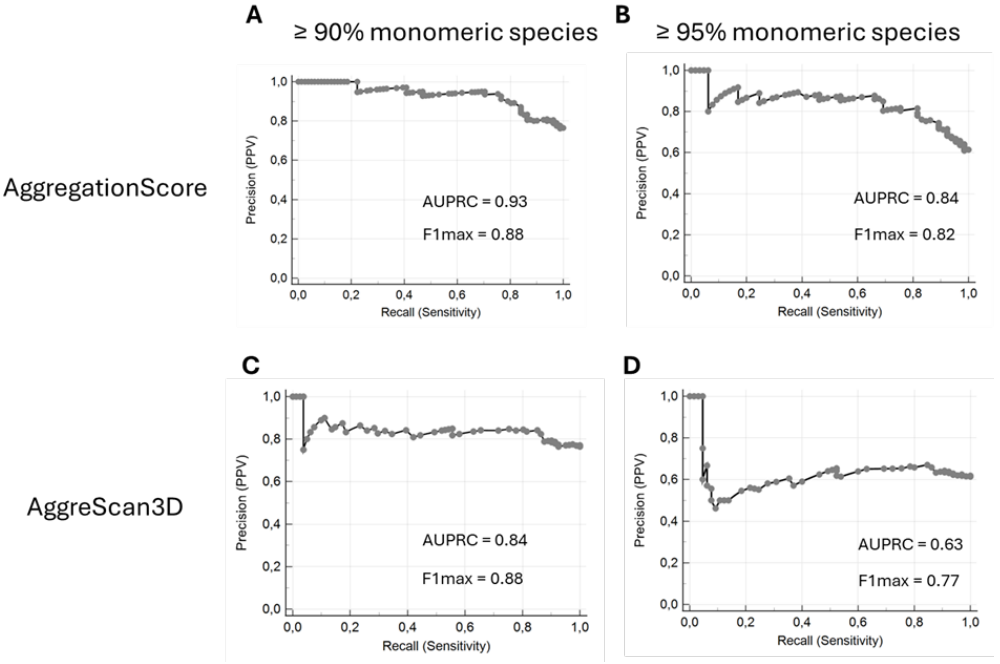

Figure S4. Precision-Recall curves (PRC) for AggregationScore and AggreScan3D. Separation into VHHs with ≥ 90% monomeric species in SEC resulted in 81 positive VHHs (P=81) and 25 negative VHHs (N=25). Separation into VHHs with ≥ 95% monomeric species in SEC resulted in 65 positive VHHs (P=65) and 41 negative VHHs (N=41). **A** PRC of AggregationScore for separation of VHHs with ≥ 90% monomeric species in SEC. An area under the Precision-Recall curve (AUPRC) of 0.93 and an F1max of 0.88 was obtained. **B** PRC of AggregationScore for separation of VHHs with ≥ 95% monomeric species in SEC. An area under the Precision-Recall curve (AUPRC) of 0.84 and an F1max of 0.82 was obtained. **C** PRC of AggreScan3D for separation of VHHs with ≥ 90% monomeric species in SEC. An area under the Precision-Recall curve (AUPRC) of 0.84 and an F1max of 0.88 was obtained. **D** PRC of AggreScan3D for separation of VHHs with ≥ 95% monomeric species in SEC. An area under the Precision-Recall curve (AUPRC) of 0.63 and an F1max of 0.77 was obtained.

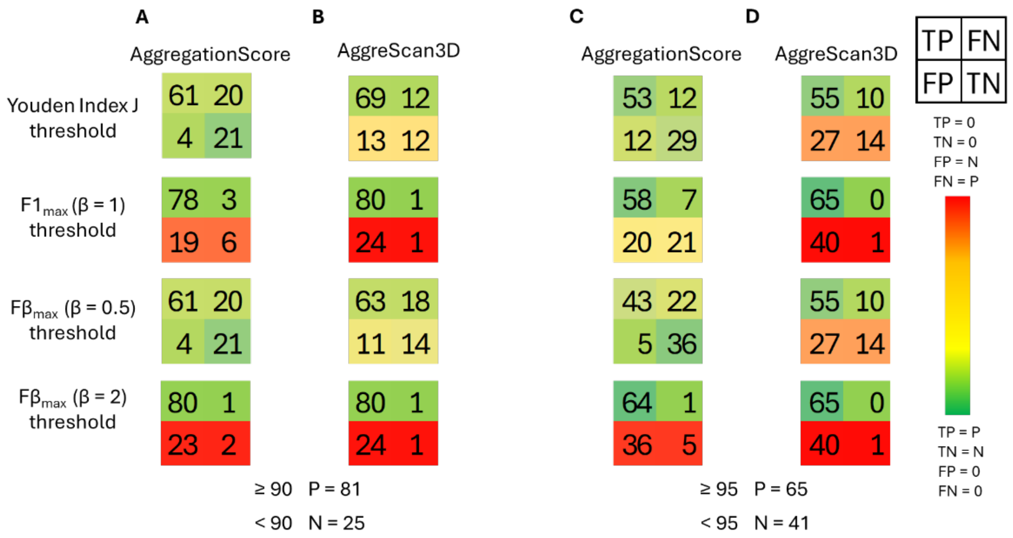

Figure S5. Confusion Matrix for different thresholds for AggregationScore and AggreScan3D. These thresholds were chosen based on the Youden index (J) from the receiver operating characteristic (ROC) curve and the maximum F1, F0.5, and F2 values from the precision–recall curve. Different β values were applied when calculating the maximum F value to adjust the focus toward recall sensitivity or precision sensitivity: β > 1 emphasizes recall, while β < 1 emphasizes precision. For a recall-oriented evaluation, a β value of 2 was selected; for a precision-oriented evaluation, a β value of 0.5 was selected. For equal weighting of precision and recall, a β value of 1 was selected. In Q1, the number of true positives (TP) is displayed; in Q2, false negatives (FN); in Q3, false positives (FP); and in Q4, true negatives (TN). The matrices are color-coded, with green representing the optimal value (TP = P; TN = N; FP = 0; FN = 0). **A** Confusion matrices for AggregationScore for separating VHHs with ≥ 90% monomeric species in SEC (P=81; N=25). By applying the threshold for Youden Index J (>0.89) an accuracy of 0.77 and a balanced accuracy of 0.8 were obtained. For the corresponding thresholds for F1max an accuracy of 0.79 and a balanced accuracy of 0.6, for F0.5max an accuracy of 0.77 and a balanced accuracy of 0.8, and for F2max an accuracy of 0.77 and a balanced accuracy of 0.53 were obtained. **B** Confusion matrices for AggreScan3D separating VHHs with ≥ 90% monomeric species in SEC (P=81; N=25). By applying the threshold for Youden Index J (≤-91) an accuracy of 0.76 and a balanced accuracy of 0.66 were obtained. For the corresponding thresholds for F1max an accuracy of 0.76 and a balanced accuracy of 0.52, for F0.5max an accuracy of 0.73 and a balanced accuracy of 0.67, and for F2max an accuracy of 0.76 and a balanced accuracy of 0.52 were obtained. **C** Confusion matrices for AggregationScore for separating VHHs with ≥ 95% monomeric species in SEC (P=65; N=41). By applying the threshold for Youden Index J (>0.89) an accuracy of 0.77 and a balanced accuracy of 0.75 were obtained. For the corresponding thresholds for F1max an accuracy of 0.75 and a balanced accuracy of 0.71, for F0.5max an accuracy of 0.75 and a balanced accuracy of 0.76, and for F2max an accuracy of 0.65 and a balanced accuracy of 0.53 were obtained. **D** Confusion matrices for AggreScan3D separating VHHs with ≥ 95% monomeric species in SEC (P=65; N=41). By applying the threshold for Youden Index J (≤-91) an accuracy of 0.65 and a balanced accuracy of 0.59 were obtained. For the corresponding thresholds for F1max an accuracy of 0.62 and a balanced accuracy of 0.51, for F0.5max an accuracy of 0.65 and a balanced accuracy of 0.59, and for F2max an accuracy of 0.62 and a balanced accuracy of 0.51 were obtained.

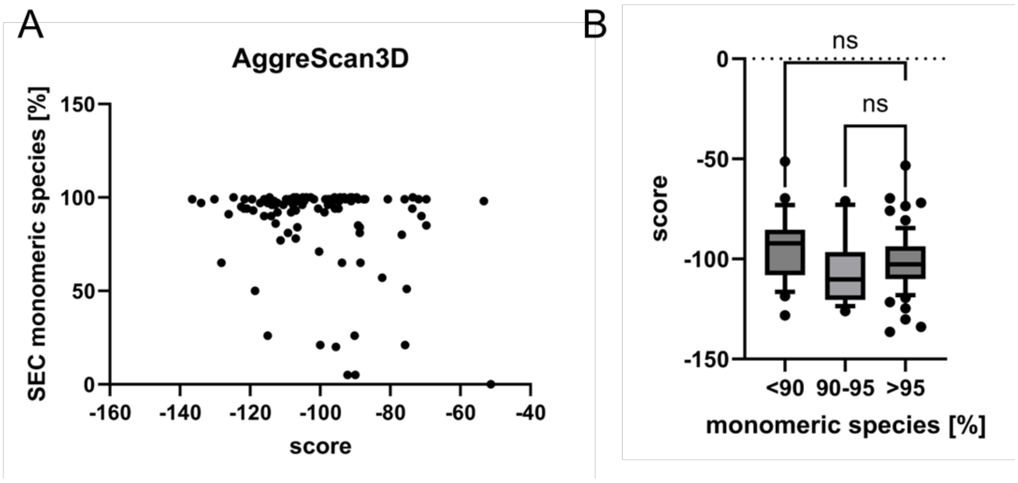

Figure S6. Analysis of the whole VHH data set byAggreScan3D 2.0. **A** Plot showing the total score on the x-axis and the monomeric species in SEC on the y-axis. **B** Box plot of 106 VHHs with Whisker showing 10-90% percentile. VHHs are grouped according to their monomeric species content in SEC: <90% (n = 25); 90–95% (n = 16); >95% (n = 65). Significance was analyzed using a one-way Krustal-Wallis- test (***** P ≤ 0.0001; *** P ≤ 0.001; ** P ≤ 0.01; P ≤ 0.05; ns P > 0.05).*

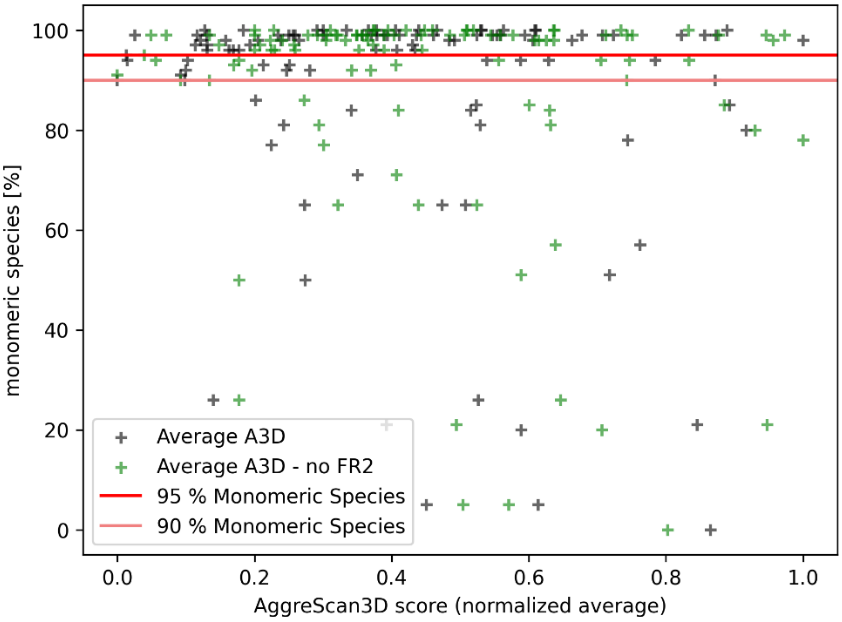

Figure S7. Relevance of the FR2 sequence patch for the prediction of VHH aggregation by AGGRESCAN3D 2.0. Aggregation behavior of 106 full-length VHH sequences correlated with aggregation propensities predicted by AGGRESCAN3D 2.0 with (black) and without (green) the FR2 region (residues 39–55), normalized using min-max normalization. Thresholds for 90% (light red) and 95% (red) monomeric species, as determined by size-exclusion chromatography (SEC), are indicated. Exclusion of the FR2 region results in an average aggregation score shift of 7.8%.

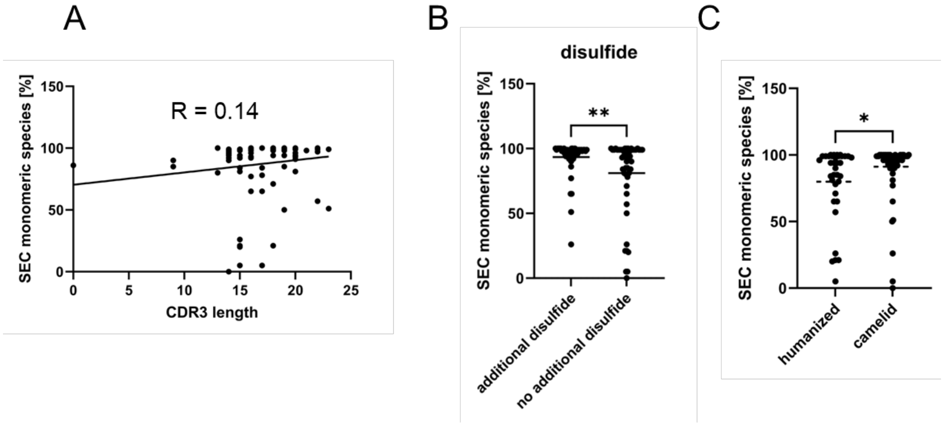

Figure S8. Impact of CDR3 length, the presence of non-canonical disulfide bridge and humanization on aggregation behavior of VHHs. A Plot showing the monomeric species in SEC on the y-axis and the CDR3 length on the x-axis. No significant correlation was observed with a Pearson coefficient of 0.14. B Scatter plot comparing the aggregation for VHHs with and without non-canonical disulfide bridge. 54 VHHs had an additional disulfide bridge and had an average of 93.4% monomeric species in SEC, whereas 52 VHHs had no additional disulfide bridge and had on average 81.1% monomeric species in SEC. C Scatter plot comparing the aggregation for VHHs that are humanized or camelid. 36 VHHs were humanized and showed on average 79.9% monomeric species in SEC, whereas 70 VHHs were not humanized and showed on average 91.2% monomeric species in SEC. Significance was analyzed using an unpaired t test (**** P ≤ 0.001; ** P ≤ 0.01; P ≤ 0.05; ns P > 0.05).*

Table S1. Origin, monomeric species measured in SEC and calculated score for ROR1 targeting VHHs.

Clone ID	origin	Monomeric species [%]	AggregationScore
VHH1	Bactrian Camel 1 immunization	77	0.68
VHH2	Bactrian Camel 1 immunization	99	1.18
VHH1_LAS	Humanization of VHH1 (IGHV3-23)	65	0.88
VHH2_LAS	Humanization of VHH2 (IGHV3-23)	99	1.04
VHH1_LVAS	Humanization of VHH1 (IGHV3-23)	100	1.45
VHH1_LAAS	Humanization of VHH1 (IGHV3-23)	96	1.18
VHH1_LVS	Humanization of VHH1 (IGHV3-23)	100	1.35
IGHV3-66-CDRVHH1	Humanization of VHH1 (IGHV3-66)	98	0.99
huVHH1	Humanization of VHH1 (IGHV3-66)	100	1.17
IGHV3-66-CDRVHH1	Humanization of VHH1 (IGHV3-64)	93	1.02

Table S2. Origin, monomeric species measured by SEC, and calculated score for VHHs targeting antigen 2.

Clone ID	origin	Monomeric species [%]	AggregationScore
1063	Bactrian Camel 3 immunization	91	2.19
1064	Bactrian Camel 3 immunization	99	1.31
1065	Bactrian Camel 3 immunization	99	1.75
1072	Bactrian Camel 3 immunization	96	2.45
1073	Bactrian Camel 3 immunization	100	1.57
1074	Bactrian Camel 3 immunization	96	1.71
1075	Bactrian Camel 3 immunization	100	2.00
1076	Bactrian Camel 3 immunization	97	1.20
1077	Bactrian Camel 3 immunization	100	1.12
C4	Bactrian Camel 3 immunization	5	0.12
dc28	Bactrian Camel 3 immunization	99	2.56
dc3	Bactrian Camel 3 immunization	99	1.57
C10	Bactrian Camel 3 immunization	99	0.56
C11	Bactrian Camel 3 immunization	99	0.11
S4	Bactrian Camel 3 immunization	50	0.53
F2	C4 humanization (IGHV3-23)	20	0.67
F4	C4 humanization (IGHV3-23)	98	0.51
G1	C4 humanization (IGHV3-23)	81	0.89
G2	C4 humanization (IGHV3-23)	84	0.65
G3	C4 humanization (IGHV3-23)	26	0.82
1346	1072 humanization (IGHV3-23)	99	1.70
1347	1072 humanization (IGHV3-23)	99	1.07

1348	1072 humanization (IGHV3-23)	99	1.93
1349	1072 humanization (IGHV3-23)	99	2.96
1350	1072 humanization (IGHV3-23)	99	1.86
1351	1072 humanization (IGHV3-23)	99	1.29
Arg103	C4 humanization (IGHV3-23)	21	0.78

Table S3. Summary of statistical metrics for separating VHHs with ≥90% monomeric species in SEC (P=81; N=25) comparing AggregationScore and AggreScan3D.

Metric	AggregationScore	AggreScan3D
AUROC	0.81	0.65
P (AUROC)	< 0.0001	0.0223
Youden Index J	0.59	0.33
Youden Index J threshold	>0.89	≤-91
Youden Index J threshold accuracy	0.77	0.76
Youden Index J threshold balanced accuracy	0.80	0.66
AUPRC	0.93	0.84
F1max	0.88	0.88
F1max threshold	> 0.48	< -52.3
F1max threshold accuracy	0.79	0.76
F1max threshold balanced accuracy	0.60	0.52
F0.5max	0.90	0.84
F0.5max threshold	> 0.89	< -94.1
F0.5max threshold accuracy	0.77	0.73
F0.5max threshold balanced accuracy	0.80	0.67
F2max	0.94	0.95
F2max threshold	> 0.27	< -52.3
F2max threshold accuracy	0.77	0.76
F2max threshold balanced accuracy	0.53	0.52

Table S4. Summary of statistical metrics for separating VHHs with ≥95% monomeric species in SEC (P=65; N=41) comparing AggregationScore and AggreScan3D.

Metric	AggregationScore	AggreScan3D
AUROC	0.79	0.53
P (AUROC)	< 0.0001	0.60
Youden Index J	0.52	0.19
Youden Index J threshold	>0.89	≤-91
Youden Index J threshold accuracy	0.77	0.65
Youden Index J threshold balanced accuracy	0.75	0.59
AUPRC	0.84	0.63
F1max	0.82	0.76
F1max threshold	> 0.78	< -52.3
F1max threshold accuracy	0.75	0.62
F1max threshold balanced accuracy	0.71	0.51
F0.5max	0.82	0.70
F0.5max threshold	> 1.06	< -90.6
F0.5max threshold accuracy	0.75	0.65
F0.5max threshold balanced accuracy	0.76	0.59
F2max	0.89	0.89
F2max threshold	> 0.42	< -52.3
F2max threshold accuracy	0.65	0.62
F2max threshold balanced accuracy	0.53	0.51
